# Concurrence Percolation Behavior in Diluted Quantum Networks

**DOI:** 10.3390/e28060590

**Published:** 2026-05-26

**Authors:** Gaogao Dong, Yili Shen, Xinqi Hu, Ruijin Du

**Affiliations:** School of Mathematical Sciences, Jiangsu University, Zhenjiang 212013, China; gago999@126.com (G.D.); yili272026@163.com (Y.S.)

**Keywords:** hierarchical scale-free, diluted, concurrence percolation theory, robustness

## Abstract

The evolution of connectivity in quantum networks under decoherence and link degradation is a central problem in quantum information, calling for further understanding of the nature of its transition during structural network degradation. By diluting each link with probability 1−f, we focus on connectivity strength transitions in diluted hierarchical scale-free quantum networks, the (u,v) flowers, which are analytically tractable through two adjustable path-length parameters, u≤v. Incorporating quantum concurrence percolation and comparing it with classical percolation, we analyze the transitions of critical thresholds for various values of *f* and *v* from analytical, numerical, and simulation perspectives. The results demonstrate that quantum percolation exhibits consistently lower critical thresholds than classical percolation, even under various topologies and dilution levels. Our work implies that quantum multipath entanglement provides an intrinsic compensatory mechanism against structural degradation and that the hierarchical scale-free topology contributes to the failure resistance and robustness of quantum networks with multipath coupling.

## 1. Introduction

Modern information infrastructures and physical networked systems are rapidly evolving toward large-scale, heterogeneous, and highly complex architectures, making their structural vulnerability under random failures, noise perturbations, or targeted attacks a central topic in network science. Previous studies have demonstrated that, in classical complex networks, randomly removing edges can induce abrupt transitions in network connectivity: a giant connected component persists below a critical occupation probability but collapses once the threshold is exceeded. Percolation theory provides a rigorous framework for characterizing such structural phase transitions. The seminal criterion of Molloy and Reed for the emergence of giant components [1], together with the work of Albert, Barabási, and Cohen, systematically analyzed the differences in network robustness under random failures and targeted attacks [2,3,4], and established dilution as a standard model for structural defects. Since then, percolation and phase-transition theory have become fundamental mathematical tools for assessing the robustness of communication networks, power grids, transportation systems, and neural networks [5,6].

Unlike classical networks, quantum networks rely on shared entanglement across distant nodes to enable long-distance quantum communication, distributed quantum computing, and quantum key distribution [7]. Experimental progress has demonstrated the feasibility of long-distance entanglement distribution and quantum communication networks, further motivating the study of their robustness under realistic conditions [8]. However, the quality of quantum links is constrained by multiple quantum effects, including decoherence, photon loss, channel noise, and the non-negligible cost of entanglement swapping. These features highlight two essential aspects of quantum network resilience: (i) structural defects (dilution) do not translate linearly into entanglement reachability, and (ii) quantum communication protocols, such as entanglement swapping, entanglement purification, and parallel-path strategies, fundamentally modify the path-level merging rules, thereby introducing nonlinear statistical behaviors that are absent in classical networks [9,10,11].

To relate entanglement distribution to classical percolation, Acín et al. introduced the Classical Entanglement Percolation (CEP) framework [12], modeling each entangled link as an occupied edge convertible into a Bell pair and requiring a fully maximally entangled path for long-distance entanglement. However, subsequent studies demonstrated that multipath structures, local operations and classical communication (LOCC), and entanglement swapping can significantly enhance end-to-end entanglement transmission, making CEP overly conservative in predicting thresholds [13,14]. In particular, Perseguers et al. showed that quantum protocols can substantially lower percolation thresholds and yield a clear ”quantum advantage,” indicating that the critical behavior of diluted quantum networks fundamentally differs from classical percolation.

To capture the cooperative enhancement of entanglement through multiple partially entangled paths, Meng and co-workers proposed Concurrence Percolation Theory (ConPT) [15,16]. ConPT uses concurrence as the basic variable and employs nonlinear series- and parallel-combination rules to quantify network-level entanglement connectivity, thereby circumventing CEP’s reliance on perfect paths. ConPT improves the accuracy of threshold predictions across a wide range of topologies, including Bethe lattices, recursive tree networks, and other renormalizable structures. Nonetheless, existing work focuses primarily on tree-like or low-cycle graphs; comprehensive analyses for more general complex topologies—those exhibiting fractal geometry, small-world behavior, or strong heterogeneity—remain limited.

Hierarchical scale-free networks constitute an important class of models that lie between regular and random structures [17]. Among them, the (u,v) flower network provides a deterministic, analytically tractable topology with exact self-similarity, tunable fractal dimension, and scale-free degree distribution. Rozenfeld and Ben-Avraham demonstrated that its recursive construction enables exact real-space renormalization and analytical determination of percolation thresholds [18,19]. Such networks appear in diverse real-world systems, including the Internet, biochemical networks, and communication infrastructures, and their hierarchical organization makes them ideal platforms for studying percolation, spreading dynamics, and quantum-state transmission.

Motivated by these considerations, this work incorporates ConPT into hierarchical fractal structures and develops a unified entanglement-percolation framework for diluted quantum networks. Using the (u,v) flower as a representative hierarchical scale-free topology, we systematically compare classical percolation and quantum entanglement percolation under dilution, and investigate how the critical threshold, giant-cluster formation, and connectivity evolve with iteration depth, fractal dimension, and dilution strength. The main contributions of this study are as follows. First, we model dilution as a multiplicative degradation of each link’s entanglement-carrying capacity, providing a resource-oriented and implementation-relevant description for quantum networks. Second, we formulate quantum series-parallel combination rules in a recursive manner consistent with the network’s self-similarity, enabling exact finite-generation solutions and controlled extrapolation to the thermodynamic limit. Third, we establish a unified cross-validation scheme combining analytical renormalization, cluster-based methods, and Monte Carlo simulations, uncovering the cooperative effects of topology, fractal dimension, and dilution on quantum percolation thresholds and failure mechanisms. This work offers a systematic theoretical foundation for understanding the robustness of large-scale quantum networks under decoherence and link degradation, and provides insights for structure optimization in future quantum-internet architectures.

## 2. Materials and Methods

### 2.1. Network Models and Their Dilution

**Figure 1:**
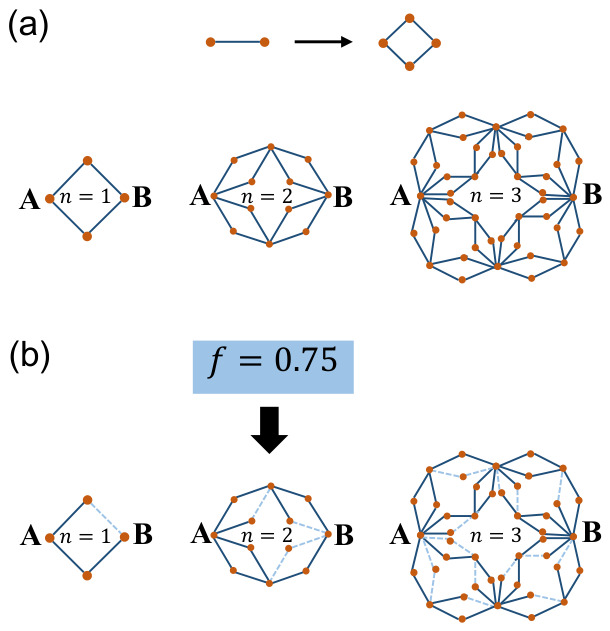
The (u,v) flower network under dilution. A random edge dilution is applied independently at each iteration generation, without inter-generation correlation, with a retention ratio f=0.75. Nodes A and B denote the hub vertices of the network.Orange dots represent network nodes. (**a**) Original (u,v) flower network. (**b**) Diluted (u,v) flower network. Solid blue lines represent remaining edges, while dashed blue lines represent removed edges.

We focus on a special type of hierarchical scale-free networks, the (u,v) flowers, which are produced recursively. Starting from a ring with (u+v) edges, the (n+1)th generation of the (u,v) flower is generated by replacing each link in the *n*th-generation network with two parallel paths between two nodes consisting of *u* and *v* links, respectively [18]. Without loss of generality, we assume 1<u≤v (Figure 1a). Given this self-similarity construction of the (u,v) flower, we can easily control its topological structure, scale, and fractal dimension $d=\frac{\ln(u+v)}{\ln u}$ by simply controlling the path length parameters *u*, *v*, and generation *n*. As a result, we obtain a series of models for the analysis of percolation connectivity on various network structures by increasing *d*, where a larger *d* represents a denser topology with more and longer intertwined paths.

On these models, it has been found that a greater number of non-shortest paths is important for enhancing the quantum network connectivity, implying higher entanglement transmission efficiency compared to its classical counterpart [16]. Following this, we are interested in the effects of structural defects and entanglement decoherence on entanglement transmission in these quantum network models. We introduce a dilution factor f∈[0,1] to characterize the retained connectivity of network links. From the classical perspective, *f* can be interpreted as the effective occupation probability of edges, reflecting the loss of connectivity caused by random failures or structural defects. From the quantum perspective, *f* represents the proportion of entanglement carrying capacity retained on each edge, which characterizes the degradation of entanglement resources induced by decoherence, photon loss, and channel noise. Therefore, as a unified effective parameter, *f* captures both structural defects and quantum noise effects. In the limiting cases, f=1 corresponds to an ideal undiluted network, while f=0 indicates a complete loss of connectivity with no possibility of entanglement transmission. Intermediate values describe the partial degradation of classical or quantum resources. Figure 1b shows an example of dilution on (2, 2) flower networks with generations from 1 to 3.

### 2.2. Measurements of Connectivity

**Figure 2:**
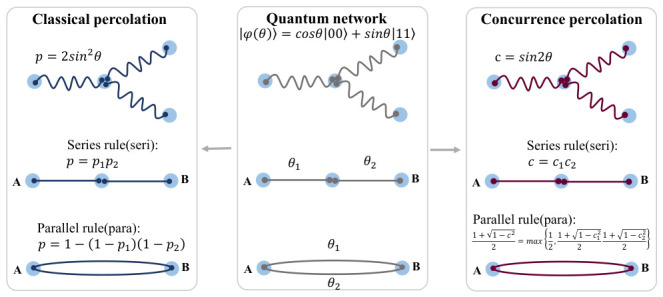
Percolation mappings of a quantum network. In a QN, a node comprises several qubits, each entangled with those in other nodes, and a link represents a partially entangled bipartite state |ψ(θ)〉 shared between two nodes (grey). Entanglement transmission across the QN can be understood based on two distinct statistical physics mappings: classical percolation (blue) versus concurrence quantum percolation (purple). Each mapping is based on its own set of series and parallel path connectivity rules, which dictate the superposition of multiple path connectivities to determine the total percolation connectivity between two distant nodes, say, Alice (node A) and Bob (node B).

To measure the strength of connectivity between two distant nodes on a quantum network, we start from the pure-state entangled quantum network consisting of qubits (nodes) and entanglement between them (edges), where each edge corresponds to a pure entangled state |ψ(θ)〉=cosθ|00〉+sinθ|11〉 with $0\leq \theta \leq \frac{\pi }{4}$. For analytical tractability, we assume that all edges correspond to identical pure entangled states; see Figure 2. Classical percolation as a traditional network science method has been applied to the entanglement transmission on quantum network based on classical series and parallel connectivity rules, which are given by $\mathrm{seri}\left(p_{1},p_{2},\ldots \right)=p_{1}p_{2}\ldots$ for series rule and $\mathrm{para}\left(p_{1},p_{2},\ldots \right)=1-\left(1-p_{1}\right)\left(1-p_{2}\right)\ldots$ for parallel rule. $p_{i}=2\sin^{2}\theta_{i}$ is the optimal conversion probability for a single link *i* while “seri” and “para” denote effective connectivity of the final path composed of serial or parallel links, respectively. This mapping to entanglement is referred to as Classical Entanglement Percolation (CEP).

However, in realistic quantum networks, operations between nodes are restricted to LOCC [20], which prevents the direct construction of optimal single-path transmission strategies. Instead, different entangled paths are not independent, and global connectivity between distant nodes can be established through the cooperative interaction of multiple paths. From a resource-theoretic perspective, this behavior originates from the non-additivity of quantum entanglement. Specifically, multiple partially entangled paths can be jointly processed under LOCC to form effective long-range entanglement, whose overall effect cannot be expressed as a linear sum of individual contributions, but is instead enhanced through nonlinear composition rules. Therefore, quantum connectivity is fundamentally governed by multipath cooperation rather than single-path optimization. Therefore, concurrence—defined as $c_{i}=\sin2\theta_{i}$ and used as a key measure of bipartite entanglement for a pure state—is applied to path connectivity in the quantum case, leading to an alternative mapping known as ConPT. In this framework, the path connectivity rules by LOCC are given by $\mathrm{seri}\left(c_{1},c_{2},\ldots \right)=c_{1}c_{2}\ldots$ for series rule and $\frac{1+\sqrt{1-\mathrm{para}{\left(c_{1},c_{2},\ldots \right)}^{2}}}{2}=\max\left\{\frac{1}{2},\;\frac{1+\sqrt{1-c_{1}^{2}}}{2}\cdot \frac{1+\sqrt{1-c_{2}^{2}}}{2}\;\ldots \right\}$ for the parallel rule [15]. Here, $\mathrm{para}{\left(c_{1},c_{2},\ldots \right)}^{2}$ denotes the effective concurrence of two parallel paths after LOCC merging; $\frac{1+\sqrt{1-c_{1}^{2}}}{2}\cdot \frac{1+\sqrt{1-c_{2}^{2}}}{2}\;\cdots$ represents the joint contribution of the two partially entangled paths; the “max” operation arises from the fact that the entanglement formation probability cannot be lower than the baseline value of $\frac{1}{2}$ corresponding to the completely unentangled state.

### 2.3. Calculations of Percolation

We denote by $C_{\mathrm{sc}}$ the quantum sponge-crossing concurrence—the equivalent concurrence between two far-apart nodes, A and B. For an infinite-size quantum network, $C_{\mathrm{sc}}$ undergoes a phase transition characterized by a sudden jump from 0 to 1 at the critical threshold $c_{\mathrm{th}}$. In contrast to the critical threshold $p_{\mathrm{th}}$ of classical sponge-crossing probability $P_{\mathrm{sc}}$, a nontrivial $c_{\mathrm{th}}$ is always lower, indicating stronger connectivity in an undiluted quantum network.

On diluted (u,v) flowers, we investigate $C_{\mathrm{sc}}$ compared to $P_{\mathrm{sc}}$ for increasing generation *n* and various values of the dilution degree *f*, using three approaches: (1) Analytically, we adopt a mean-field effective approximation, in which the stochastic bond occupation is replaced by its average effective connectivity contribution, leading to the mapping Q→fQ, where fQ represents the average effective connectivity retained after random bond dilution under the mean-field approximation. Therefore, the recursive equations describe the average effective connectivity induced by random dilution. Based on the series-parallel composition rules, the renormalization group function is constructed as follows:

(1) $$R\left(Q\right)=\mathrm{para}(\mathrm{seri}(\overset{U}{\overset{︷}{fQ,fQ,\ldots ,fQ}}),\;\mathrm{seri}(\overset{V}{\overset{︷}{fQ,fQ,\ldots ,fQ}}))$$

where the initial value of *Q* is *p* in the classical case and *c* in the quantum case. Critical thresholds are determined by solving for the fixed points $Q^{*}$ from $R\left(Q^{*}\right)=Q^{*}$. The disappearance of a nontrivial fixed point leads to the absence of a threshold. (2) Numerically, $C_{\mathrm{sc}}\left(n\right)$ and $P_{\mathrm{sc}}\left(n\right)$ are computed by recursively applying Equation (1) a finite number of times, showing their convergence behavior as n→∞. (3) In the simulation, the Monte Carlo method is naturally applied to classical percolation, where a 1−f fraction of the edges is randomly removed from an original complete (u,v) flower network (Figure 1). Percolation on the diluted network is estimated through statistical averaging, with error bars included to characterize finite-sample fluctuations and to assess consistency with the analytical and numerical results. Open boundary conditions are employed in the Monte Carlo simulations to measure the connectivity between two specified boundary nodes, with each simulation repeated 1000 times under the same parameter settings. The critical threshold in this work is rigorously determined by the nontrivial fixed point of the renormalization equation, satisfying Equation (1). In addition, numerical estimates of the critical point are obtained from the transition of connectivity measures from zero to nonzero values in the thermodynamic limit. The Monte Carlo simulations provide a microscopic stochastic realization of bond dilution, while the renormalization approach gives a coarse-grained description of its average connectivity effect.

In addition, we denote the quantum order parameter by $C_{\infty }$ by analogy with the classical order parameter $P_{\infty }$, which represents the fraction of nodes that belong to the giant component, and define it as the concurrence with which a node can participate in long-range quantum entanglement transmission in a quantum network. For original (u,v) flowers, $Q_{\infty }$ remains zero for all $q<q_{\mathrm{th}}$ and continuously decreases as $q\to q_{\mathrm{th}}^{+}$, following the power law relation $Q_{\infty }∼{\left(q-q_{\mathrm{th}}\right)}^{\beta_{q}}$ such that a second-order phase transition occurs near $q_{\mathrm{th}}$, where *q* denotes the probability *p* for classical percolation and the concurrence *c* for quantum percolation. Consequently, $\beta_{c}\neq \beta_{p}$, indicating that concurrence percolation belongs to a different universality class from classical percolation [16]. This distinction arises from the global series and parallel connectivity rules of concurrence percolation that strengthen the connection between A and B.

**Figure 3:**
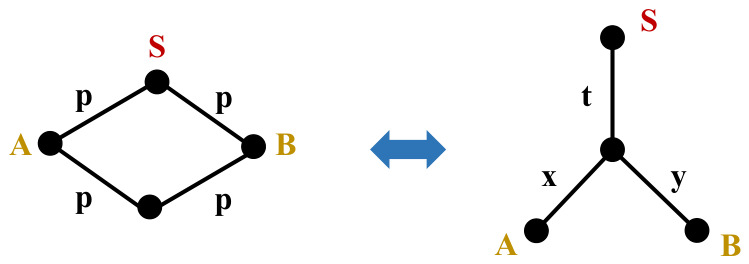
Schematic diagram of the star–mesh transformation. Black dots represent network nodes. A and B are the terminal nodes, *S* is the central star node. *p* denotes the edge parameters in the mesh structure, and *t*, *x*, *y* denote the transformed parameters in the star structure.

Similarly, we are interested in the critical behavior of order parameters in diluted (u,v) flowers with various values of *f*. Concurrence percolation $C_{\infty }$, which is difficult to explain using a cluster-based framework, can be approximated using star–mesh transformation simulations [16], as shown schematically in Figure 3.

We solve the connectivity between a non-hub node *s* with degree 2 and A or B by recursively transforming the original network into a star topology *S* where *s*, A, and B are connected with an added root node *r*. This process begins by equating the first generation to such a star. For generation *n*, after one of the copies of the (n−1)th-generation (u,v) flower has been transformed into *S*, we coarse-grain the network into a star *S* through a group of iterative functions based on series and parallel rules:

(2) $$\left\{ \begin{array}{l} \left\{ \begin{array}{l} \mathrm{seri}(x^{\prime },t^{\prime })=\mathrm{seri}(t,\mathrm{para}(\mathrm{seri}(fx,\overset{a}{\overbrace{Q,Q,\cdots ,Q}}),\mathrm{seri}(fy,\overset{u+v-1-a}{\overbrace{Q,Q,\cdots ,Q}})))\\ \mathrm{seri}(y^{\prime },t^{\prime })=\mathrm{seri}(t,\mathrm{para}(\mathrm{seri}(fx,\overset{u-1-a}{\overbrace{Q,Q,\cdots ,Q}}),\mathrm{seri}(fy,\overset{v+a}{\overbrace{Q,Q,\cdots ,Q}})))\\ \mathrm{seri}(x^{\prime },y^{\prime })=\mathrm{para}(\mathrm{seri}(\overset{u}{\overbrace{Q,Q,\cdots ,Q}}),\mathrm{seri}(\overset{v}{\overbrace{Q,Q,\cdots ,Q}})) \end{array}\right.\\ \left\{ \begin{array}{l} \mathrm{seri}(x^{\prime },t^{\prime })=\mathrm{seri}(t,\mathrm{para}(\mathrm{seri}(fx,\overset{b}{\overbrace{Q,Q,\cdots ,Q}}),\mathrm{seri}(fy,\overset{u+v-1-b}{\overbrace{Q,Q,\cdots ,Q}})))\\ \mathrm{seri}(y^{\prime },t^{\prime })=\mathrm{seri}(t,\mathrm{para}(\mathrm{seri}(fx,\overset{v-1-b}{\overbrace{Q,Q,\cdots ,Q}}),\mathrm{seri}(fy,\overset{u+b}{\overbrace{Q,Q,\cdots ,Q}})))\\ \mathrm{seri}(x^{\prime },y^{\prime })=\mathrm{para}(\mathrm{seri}(\overset{u}{\overbrace{Q,Q,\cdots ,Q}}),\mathrm{seri}(\overset{v}{\overbrace{Q,Q,\cdots ,Q}})) \end{array}\right. \end{array}\right.$$

where a=0,1,2,⋯,u−1 and b=0,1,2,⋯,v−1. (x,y,t) represents the equivalent weights on the edges (A−r,B−r,s−r) in *S*, which is transformed from the (u,v) flower of the (n−1)th generation, while $\left(x^{\prime },y^{\prime },t^{\prime }\right)$ are the weights corresponding to the *n*th-generation network. Naturally, the initial values of (x,y,t) are (1,q,1), which come from a single link between A and B with connectivity q=p for classical percolation and q=c for concurrence percolation. From generation 2 onward, we assume $x^{\prime }=y^{\prime }$ given the symmetric network structure. Considering all (u+v) choices of node *s* at each recursion step of the equation group, we take the uniform average of the (u+v) values of $\left(x^{\prime },y^{\prime },t^{\prime }\right)$ as (x,y,t) for the next recursion. In the final step, $Q_{\infty }=\mathrm{seri}\left(t^{\prime },\mathrm{para}\left(x^{\prime },y^{\prime }\right)\right)$ gives an approximation of the order parameter $C_{\infty }$ or $P_{\infty }$.

Furthermore, we compare the classical outcomes of $P_{\infty }$ with corresponding results from Monte Carlo simulations and exact cluster measurements, which have been studied [17], to validate the reliability of the above approach. In Monte Carlo simulations, edges in the original network are randomly removed at a ratio of (1−f). A Bernoulli experiment is used to estimate the average fraction of nodes belonging to the infinite cluster to obtain $P_{\infty }$.

## 3. Results

### 3.1. Concurrence $C_{\mathrm{sc}}$ Under Dilution Compared to Classical $P_{\mathrm{sc}}$

**Figure 4:**
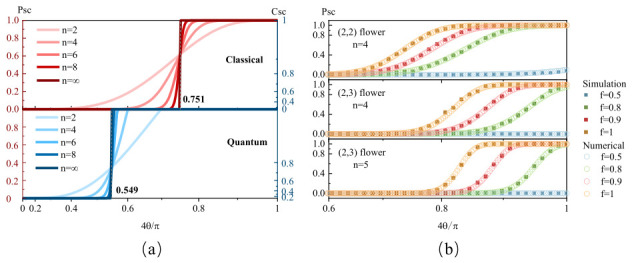
Sponge-crossing percolation on (u,v) flower networks. (**a**) Finite-size analysis of $P_{\mathrm{sc}}$ and $C_{\mathrm{sc}}$ with n=2,4,6,8; both curves tend toward the vertical lines, with the darkest colors corresponding to n=∞ in the undiluted (2, 2) flower. Red and blue colors demonstrate classical and concurrence percolation, respectively. Larger *n* corresponds to darker colors. The dashed lines and the corresponding values represent the critical thresholds. (**b**) Comparison of $P_{\mathrm{sc}}$ obtained from the numerically calculated recursive renormalization function (solid squares) and Monte Carlo simulations (hollow circles) in randomly diluted (u,v) flowers with f=0.5,0.8,0.9,1. In the top panel, u=v=2 and n=4. In the middle panel, u=2,v=3, and n=4. In the bottom panel, u=2,v=3, and n=5.

For the trivial case f=1, the network remains undiluted. The $P_{\mathrm{sc}}$ and $C_{\mathrm{sc}}$ values for finite generations n=2,4,6,8 and for the infinite system size on the (2,2) flower network are calculated using Equation (1) (Figure 4). The results show that, as *n* increases, the smooth curves that denote numerical solutions of finite-size systems approach the fixed points obtained in the infinite-system limit (indicated by vertical lines), exhibiting continuous second-order phase transitions for both quantum and classical percolation. In both cases, two unstable trivial fixed points at 0 and 1 are observed, together with a stable fixed point corresponding to the critical threshold $0<x_{\mathrm{th}}<1$. Under the unified angular representation, the concurrence threshold $\frac{4\theta_{\mathrm{th}}}{\pi }\approx 0.549\left(4\right)$ is significantly lower than the classical threshold $\frac{4\theta_{\mathrm{th}}}{\pi }\approx 0.751\left(3\right)$ (Figure 4a).

Based on this foundation, we extend the analysis to the general case 0≤f≤1; the effect of random dilution is incorporated into the recursive equations through the effective connectivity mapping (Q→fQ). This equivalence enables the derivation of Equation (1) with variable *f*, providing a self-consistent framework for computing the numerical solutions of $C_{\mathrm{sc}}$ and $P_{\mathrm{sc}}$. To validate this analytical approach, we compare its predictions for classical percolation (hollow circles) against results obtained from extensive Monte Carlo simulations (solid squares) in Figure 4b. Each panel for a fixed *n* and a fixed *v* shows that the two methods exhibit excellent agreement, with discrepancies falling within statistical uncertainty over f=0.5,0.8,0.9,1 above the dilution threshold $f_{th}$ of classical percolation. From this calculation, we obtain the formula for the $f_{\mathrm{th}}$ as a function of the independent variables $p^{\left(n-1\right)}$, *u*, and *v*:

(3) $$f_{\mathrm{th}}=f_{\max}\;\left(p^{\left(n-1\right)},u,v\right)=\min\left(\frac{x^{*}}{p^{\left(n-1\right)}},1\right),$$

where $x^{*}$ is the unique positive real solution to the equation $x^{u+v}-x^{u}-x^{v}=0$, and $p^{\left(n-1\right)}$ is the percolation threshold of the network after n−1 rounds of dilution. As *f* falls below $f_{th}$, $P_{\mathrm{sc}}$ drops to zero with $0\leq \theta \leq \frac{\pi }{4}$. Correspondingly, the formula for calculating the quantum percolation threshold $f_{\mathrm{th}}$ with respect to $c^{\left(n-1\right)}$, *u* and *v* is given by:

(4) $$\sqrt{1-{\left[\left|{\left(\frac{1+\sqrt{1-{\left(f_{\mathrm{th}}c^{\left(n-1\right)}\right)}^{2}}}{2}\right)}^{u}-\frac{1}{2}\right|+{\left(\frac{1+\sqrt{1-{\left(f_{\mathrm{th}}c^{\left(n-1\right)}\right)}^{2}}}{2}\right)}^{u}-\frac{1}{2}\right]}^{2}}=1$$

where $c^{\left(n-1\right)}$ is the percolation threshold of the network after n−1 rounds of dilution.

We further validate the renormalization results through comparison with Monte Carlo simulations from the perspective of varying path-length scale and network size, by examining n=4,5 and v=2,3 as representative cases and investigating $P_{\mathrm{sc}}$ at selected values of *f*. The top and middle panels, plotted with identical colors (fixed *f*) for n=4, illustrate the connectivity between two boundary nodes in classical (u,v) networks as *v* varies. The dependence on different *n* for the classical (2,3) flower is shown by consistent symbols across the middle and bottom panels (e.g., red solid squares denote f=0.9). Both analyses demonstrate the consistency of the analytical approach.

**Figure 5:**
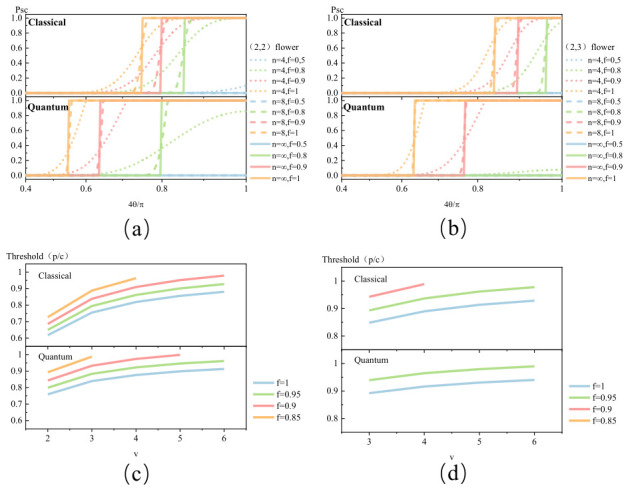
Comparison of results for diluted (u,v) flowers. (**a**) Comparison of classical and quantum results for the (2, 2) flower at different iteration levels and dilution conditions. (**b**) Comparison of classical and quantum results for the (2, 3) flower at different iteration levels and dilution conditions. (**c**) Comparison of classical and quantum thresholds for the (2, 2) flower under different dilution conditions. (**d**) Comparison of classical and quantum thresholds for the (2, 3) flower under different dilution conditions.

Therefore, quantum concurrence percolation $C_{\mathrm{sc}}$, compared with $P_{\mathrm{sc}}$ in diluted (u,v) flower networks, can be systematically analyzed using the renormalization given in Equation (1), focusing on the evolution of their critical thresholds $q_{\mathrm{th}}\left(f\right)$ under the coupled influence of generation *n* and dilution level *f*, as shown in Figure 5a,b. For each fixed f=0.8,0.9,1 and v=2 or 3, both percolation processes converge to a nontrivial threshold as *n* increases from 4 (dotted lines), through 8 (dashed lines), to *∞* (solid lines). However, for the lower value f=0.5 (blue lines), both percolation curves tend to 0, and their thresholds disappear. Thus, for certain percolation types and values of the network topology parameter *v*, there is a dilution threshold $f_{\mathrm{th}}$. Above this threshold, as *f* increases from 0.8 (green lines), through 0.9 (red lines), to 1 (orange lines), the critical thresholds (solid lines) decrease in both the classical and quantum cases. In addition, the results show that the lower the value of *f* is, the faster $c_{\mathrm{th}}$ grows relative to $p_{\mathrm{th}}$ and the later the concurrence saturation point (the smallest value of link concurrence that satisfies $C_{\mathrm{sc}}=1$) emerges for a given *n*, indicating a pronounced enhancement of quantum connectivity.

This suggests that, in n→∞, the percolation probability remains zero below the critical threshold and discontinuously jumps to unity once the threshold is reached. Therefore, it is of particular significance to determine the corresponding percolation thresholds, as shown in Figure 5c,d and Table 1.

For infinite-dimensional networks, the system approaches the thermodynamic limit. As *v* increases, the number and diversity of available paths grow, enhancing the effect of multipath coupling. This allows quantum percolation to establish long-range connectivity through the cooperative contribution of multiple partially entangled paths, leading to an increased advantage over classical percolation. This requires a higher occupancy fraction to achieve global connectivity, weakens the local connectivity probability, and consequently delays the overall percolation behavior. Dilution and topology jointly regulate the critical percolation behavior, while quantum mechanisms significantly postpone the system’s failure threshold.

### 3.2. Concurrence $C_{\infty }$ Under Dilution Compared to Classical $P_{\infty }$

To more accurately characterize the critical behavior of the system, the study employs the star–mesh transform, cluster method, and Monte Carlo simulations to obtain equivalent analytical results for the percolation probabilities $P_{\infty }$ and $C_{\infty }$ in the diluted (2, 2) flower network.

**Figure 6:**
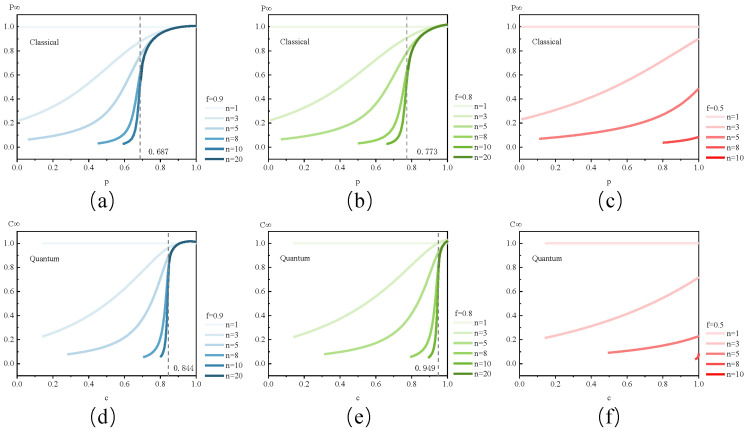
Comparison of $P_{\infty }$ and $C_{\infty }$ for diluted (u,v) flowers using star–mesh transformations. The dashed lines and the corresponding values represent the critical thresholds. (**a**–**c**) Classical percolation in the (2, 2) flower at dilution levels f=0.9, f=0.8, and f=0.5 for different iteration orders. (**d**–**f**) Quantum percolation in the (2, 2) flower at dilution levels f=0.9, f=0.8, and f=0.5 for different iteration orders.

**Figure 7:**
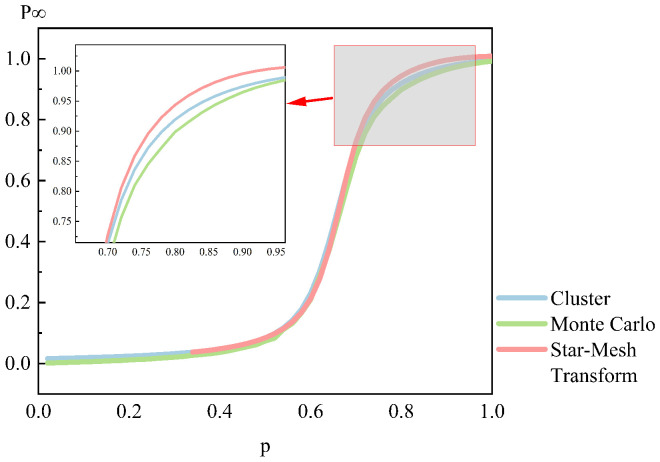
Case study of the (2,2) flower, n=6, f=0.9. The figure presents the deviations between the results obtained using the three approaches.

The (u,v) flower network is transformed into a more computationally tractable equivalent structure using the star–mesh transform, and $P_{\infty }$ and $C_{\infty }$ are obtained by combining series and parallel rules. The results demonstrate that both the increase in dilution level and the rise in network structural complexity lead to a decrease in the percolation threshold. The variations in the classical and quantum systems remain largely consistent, and both exhibit a rapid collapse in connectivity once a certain threshold is reached, supporting a unified descriptive framework for the two percolation mechanisms, as shown in Figure 6. Furthermore, using the cases of n=6 and f=0.9, a comparison of star–mesh transform (red lines), cluster (blue lines), and Monte Carlo (green lines) applied to the diluted (2, 2) flower shows that the results are highly consistent. The numerical error is controlled within 0.017. This confirms the accuracy of the analytical derivations and the reliability of the numerical simulations, as shown in Figure 7. The cluster method captures local connection probabilities, the Monte Carlo approach provides statistical validation, and the star–mesh method offers a global analytical approximation. Together, these three methods mutually reinforce each other and form a complete multiscale framework for percolation analysis.

Moreover, from Figure 6, it is observed that the percolation threshold tends to stabilize as the structural complexity of the network increases. Both $P_{\infty }$ and $C_{\infty }$ exhibit a rapid transition from zero to finite values near the critical point, indicating a continuous phase transition. The critical point corresponds to the inflection point where the curves change from a flat regime to a rapidly increasing regime. Moreover, the quantum curves transition at lower parameter values compared to the classical ones, confirming the lower critical threshold of quantum percolation. This behavior originates from the fundamental difference in connectivity mechanisms: while classical percolation relies on independent path probabilities, quantum percolation allows multiple partially entangled paths to cooperatively establish connectivity via nonlinear composition under LOCC, enabling percolation at lower parameter values. For u=2, a comparison of the thresholds of $P_{\mathrm{sc}}$ and $C_{\mathrm{sc}}$ across different values of *v* and dilution levels reveals that the corresponding thresholds of $P_{\infty }$ and $C_{\infty }$ coincide with them. The specific values are presented in Table 2 and Table 3. Therefore, $P_{\infty }$ and $C_{\infty }$, as well as $P_{\mathrm{sc}}$ and $C_{\mathrm{sc}}$, exhibit the same variation trend. As *f* decreases, the curves of $P_{\infty }$ and $C_{\infty }$ shift to larger angles θ; both the size of the network and the number of iterations lead to a rapid reduction in effective connectivity, and when *f* falls below a certain value, the effective connectivity of the entire network drops to zero, resulting in network collapse. This demonstrates the intrinsic consistency between the macroscopic percolation limit and local critical behavior, reflecting the critical self-consistency of quantum and classical percolation in hierarchical networks: the critical threshold not only determines whether a spanning cluster exists but simultaneously sets the conditions for the formation of quantum-entanglement connectivity. Overall, these results unify macroscopic critical behavior, topological parameters, and microscopic coupling mechanisms, providing a systematic framework for studying the robustness and percolation dynamics of diluted quantum networks.

## 4. Discussion

While the present framework captures the essential features of entanglement percolation in hierarchical networks, the adopted link dilution remains an idealized approximation. Although analytically convenient, it limits the description of realistic noise effects, such as continuous variations in entanglement, decoherence, and temporal fluctuations. Incorporating distributed link qualities, time-dependent noise, and correlated failures, as well as extending the framework to dynamical scenarios with entanglement generation, consumption, and adaptive routing, would improve physical realism.

Nevertheless, the results provide a robust theoretical baseline and highlight the key role of multipath entanglement in enhancing network resilience.

## 5. Conclusions

This work, based on ConPT, systematically investigates classical and quantum percolation in diluted hierarchical scale-free (u,v) flower networks. The results show that, under varying topological structures and dilution levels, quantum percolation consistently exhibits superior connectivity compared to classical percolation, with a significantly lower critical threshold. This advantage originates from the intrinsic robustness provided by multipath entanglement superposition. From a topological perspective, the fractal dimension plays a crucial role in regulating critical behavior. As the fractal dimension increases, both classical and quantum percolation thresholds rise; however, the increase in the quantum threshold is noticeably slower and tends to saturate in higher-dimensional structures. This indicates that quantum entanglement enables effective connectivity through path redundancy, rather than relying on a single critical path, thereby enhancing resilience against structural damage in complex networks. In terms of critical behavior, classical and quantum percolation exhibit fundamental differences. Classical percolation is primarily governed by the occupation probability along shortest paths, whereas quantum percolation follows a nonlinear parallel composition rule, allowing multiple partially entangled paths to be coherently combined into effective connections. As a result, quantum percolation displays a sharper transition near criticality and a slower breakdown under stronger dilution, further demonstrating its robustness in degraded environments. Moreover, the high degree of agreement among different methods over a wide parameter range confirms that the ConPT framework can reliably capture multiscale connectivity in hierarchical networks. Overall, this work uncovers the underlying principles of entanglement percolation in fractal quantum networks and clarifies the mechanism underlying the evolution of quantum connectivity under progressive degradation of link strength induced by decoherence and structural defects, providing a systematic theoretical framework for quantum information transport in complex topologies.

## Figures and Tables

**Table 1:** Percolation thresholds at d=2 for different combinations of *u*, *v* and dilution levels.

		Classical				Quantum			
u	v	f=1	f=0.95	f=0.9	f=0.85	f=1	f=0.95	f=0.9	f=0.85
2	2	0.618034	0.650562	0.686704	0.727099	0.759471	0.799443	0.843857	0.893495
3	6	0.928739	0.977620	∖	∖	0.940425	0.989922	∖	∖
4	12	0.975279	∖	∖	∖	0.973191	∖	∖	∖
5	20	0.988619	∖	∖	∖	0.983667	∖	∖	∖

Note: The symbol ∖ indicates that the threshold does not exist in this case, i.e., no 0-to-1 transition process occurs.

**Table 2:** Classical percolation thresholds $P_{th}$ for different values of *v* at u=2 under varying dilution levels.

*v*	f=1	f=0.95	f=0.9	f=0.85	f=0.8
2	0.618034	0.650562	0.686704	0.727099	0.772543
3	0.754878	0.794608	0.838753	0.888091	0.943598
4	0.819173	0.862287	0.910192	0.963732	∖
5	0.856675	0.901763	0.951861	∖	∖
6	0.881271	0.927654	0.979191	∖	∖

Note: The symbol ∖ indicates that the threshold does not exist in this case, i.e., no 0-to-1 transition process occurs.

**Table 3:** Quantum percolation thresholds $c_{th}$ for different values of *v* at u=2 under varying dilution levels.

*v*	f=1	f=0.95	f=0.9	f=0.85	f=0.8
2	0.759471	0.799443	0.843857	0.893495	0.949339
3	0.839840	0.884042	0.933156	0.988047	∖
4	0.876897	0.923049	0.974330	∖	∖
5	0.898786	0.946091	0.998651	∖	∖
6	0.913424	0.961499	∖	∖	∖

Note: The symbol ∖ indicates that the threshold does not exist in this case, i.e., no 0-to-1 transition process occurs.

## Data Availability

No new data were created or analyzed in this study; data sharing is not applicable.

## Abbreviations

The following abbreviations are used in this manuscript:

QN	Quantum Network
ConPT	Concurrence Percolation Theory
LOCC	Local Operations and Classical Communication
